# Clean subglacial access: prospects for future deep hot-water drilling

**DOI:** 10.1098/rsta.2014.0304

**Published:** 2016-01-28

**Authors:** Keith Makinson, David Pearce, Dominic A. Hodgson, Michael J. Bentley, Andrew M. Smith, Martyn Tranter, Mike Rose, Neil Ross, Matt Mowlem, John Parnell, Martin J. Siegert

**Affiliations:** 1 British Antarctic Survey, Natural Environment Research Council, High Cross, Madingley Road, Cambridge CB3 0ET, UK; 2 Faculty of Health and Life Sciences, University of Northumbria, Ellison Building, Newcastle upon Tyne NE1 8ST, UK; 3 Department of Geography, Durham University, Lower Mountjoy, South Road, Durham DH1 3LE, UK; 4 School of Geographical Sciences, University of Bristol, University Road, Bristol BS8 1SS, UK; 5 School of Geography, Politics and Sociology, Newcastle University, Newcastle Upon Tyne NE1 7RU, UK; 6 National Oceanography Centre, University of Southampton Waterfront Campus, European Way, Southampton SO14 3ZH, UK; 7 School of Geosciences, University of Aberdeen, King's College, Aberdeen AB24 3UE, UK; 8 Grantham Institute and Department of Earth Science and Engineering, Imperial College London, South Kensington SW7 2AZ, UK

**Keywords:** deep hot-water drilling, subglacial environment, clean access, environmental stewardship

## Abstract

Accessing and sampling subglacial environments deep beneath the Antarctic Ice Sheet presents several challenges to existing drilling technologies. With over half of the ice sheet believed to be resting on a wet bed, drilling down to this environment must conform to international agreements on environmental stewardship and protection, making clean hot-water drilling the most viable option. Such a drill, and its water recovery system, must be capable of accessing significantly greater ice depths than previous hot-water drills, and remain fully operational after connecting with the basal hydrological system. The Subglacial Lake Ellsworth (SLE) project developed a comprehensive plan for deep (greater than 3000 m) subglacial lake research, involving the design and development of a clean deep-ice hot-water drill. However, during fieldwork in December 2012 drilling was halted after a succession of equipment issues culminated in a failure to link with a subsurface cavity and abandonment of the access holes. The lessons learned from this experience are presented here. Combining knowledge gained from these lessons with experience from other hot-water drilling programmes, and recent field testing, we describe the most viable technical options and operational procedures for future clean entry into SLE and other deep subglacial access targets.

## 1. Introduction

The subglacial environment beneath ice sheets remains almost entirely unexplored in terms of direct measurement and sampling. Understanding the geological, microbiological, geochemical and hydrological aspects of this environment is of significant scientific interest and, ultimately, requires direct access for the deployment of scientific instruments and recovery of samples, while maintaining the integrity of both samples and the subglacial system under investigation. Accessing the base of deep ice sheets cleanly involves considerable technical and logistical challenges, especially where water is present at the bed.

While the occurrence of liquid water in the form of lakes beneath the Antarctic Ice Sheet has been known for over 40 years, it has only recently become clear how widespread such water is [1]. About 55% of the Antarctic Ice Sheet is likely to be underlain by water, occurring where the background geothermal heat flow of 40–70 mW m^−2^ and frictional heating through ice flow is sufficient to raise basal ice temperatures to the pressure melting point [2,3]. Widespread subglacial melting leads to a thin melt-water film over large areas, ultimately producing networks of subglacial drainage and storage of water within subglacial lakes of varying sizes (from sub km to greater than 200 km in length). Rather than being stable and isolated hydrological features, satellite observations over the past decade clearly show that many subglacial lakes are connected and form part of an extensive and dynamic hydrological system controlled by basal topography, originating from the ice sheet interior and radiating out to its margins [4,5,6]. As scientific interest in accessing subglacial environments continued to grow over the last 20 years, it led the Scientific Committee on Antarctic Research (SCAR) to issue a formal Code of Conduct on the exploration of subglacial aquatic environments, adopted at the XXXIV Antarctic Treaty Consultative Meeting (Buenos Aires, 2011), acknowledging subglacial systems as pristine environments of significant potential scientific value, which must be preserved during access experiments.

Access to the ice sheet base can be gained through various drilling techniques [7], all of which are logistically and technically challenging to implement. Ice-core drilling has been used to reach great depths in ice sheets on several occasions, usually after several successive seasons of drilling. Ice coring has been used occasionally to penetrate the ice base, into both dry and wet beds. In early 2012, a mechanical ice coring system was used to drill into Subglacial Lake Vostok through approximately 3770 m of ice, with lake water rising hundreds of metres into the 137 mm diameter hole. At a later date the frozen lake water was cored and sampled [8]. This method of lake access and sampling was then repeated at the same site in early 2015 [9]. An alternative to ‘classic’ ice-core drilling has been proposed by the US Rapid Access Ice Drill (RAID) programme, currently under construction, which is designed to penetrate up to 3300 m of ice in only 200 h, and is based on a modified industry standard diamond rock drilling and coring system. However, any deep-ice drilling system requires a drilling fluid, to ensure the drill hole remains open. Typically, a hydrocarbon-based fluid is used, with a density close to that of the ice to prevent the overburden pressure causing borehole closure. Traditional drill fluids host abundant microbial life and, if used to access the ice sheet bed, lead to a high probability of biological and chemical contamination of both the subglacial environment and samples retrieved from it. The use of such fluid makes ice-core drilling incompatible with the SCAR code of conduct in areas where basal water may be present.

With basal drainage networks known to evolve over timescales of days to centuries, and models of basal thermal conditions often differing in many details [2,3], determining the physical components of subglacial water flow with precision remains difficult. This uncertainty is reflected in the SCAR code of conduct which states ‘Unless there is site-specific evidence to the contrary, drilling to the base of Antarctic ice sheets should assume that the basal ice is underlain by liquid water, and that this water forms part of a subglacial drainage network requiring a high level of environmental protection’. Hence, alternative clean drilling techniques, compared with those currently available, are required across much of Antarctica where the basal ice temperature is at or is close to the melting point.

Hot-water drills (HWDs), with their inherent speed, offer the only feasible alternative for accessing deep aquatic subglacial environments, using and recycling multiple times the water melted from the surrounding ice sheet to create access holes that are water filled. As the drilling water passes through the drilling system it can be ultra-filtered, UV-treated and pasteurized before being used to melt the access hole, and so can contain significantly less microbial and particulate content than the surrounding ice. This drill fluid, and hence hot-water drilling, is fully compliant with the SCAR Code of Conduct on subglacial exploration. Such drills are known as Clean Hot Water Drills (CHWDs).

**Figure 1.**
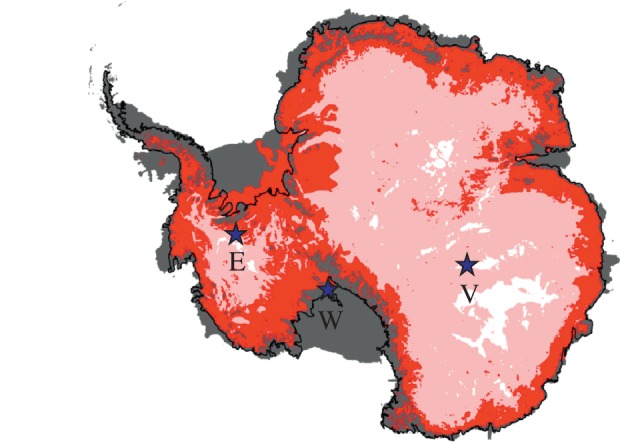
Map showing Antarctic ice thickness in depth bands corresponding with current and future hot-water drill (HWD) systems: 0–800 m (grey) corresponding to present clean HWDs; 800–2000 m (red) current HWDs that are not clean; 2000–3500 m (pink) areas that could be accessed with a modified SLE clean HWD. The stars indicate the locations of subglacial lakes Ellsworth (E), Whillians (W) and Vostok (V).

To date, the deepest successful use of a CHWD is to approximately 800 m by the US WISSARD project, melting a 60 cm diameter access hole into Subglacial Lake Whillans (figure 1) in late January 2013. The WISSARD CHWD, consists of 26 ski-mounted modules containing the drill, accompanying science laboratories and camp infrastructure, and weighs more than 230 T, requiring substantial logistical support [10]. With relatively minor modifications, the drill could have a 2000 m depth capability by substituting a smaller 19 mm (0.75′′) bore drill hose, making smaller holes and using smaller instruments. Alternatively, using multiple hose reels to accommodate lengths of 32 mm (1.25′′) hose and expanding the heating and pumping plant, a 2000 m capability could also be achieved [11].

By contrast, the Subglacial Lake Ellsworth (SLE) CHWD and its entire camp infrastructure were transported by three tractors and eight sleds, with the traverse and drill fuel requiring additional transport. However, the full deep drilling capabilities (greater than 3000 m) of this CHWD remain untested after technical issues halted drilling at the SLE site in West Antarctica (figure 1) in December 2012. Notwithstanding this setback, much of the SLE CHWD design remains appropriate to its required function, and a formal programme failure review board (FRB) has identified the specific re-engineering required to provide a reliable and portable CHWD system.

Currently, the state of the art for deep HWD technology is represented by the massive (greater than 600 T) Enhanced Hot-Water Drill (EHWD) used to build the IceCube neutrino observatory array at the South Pole over seven consecutive field seasons [12]. While the drill created 86 holes, 60 cm in diameter, to depths of 2500 m, its size and lack of clean fluid technology mean that it cannot feasibly be used more widely. However, the engineering investment over its years of operation, and the retained engineering experience resulted in significant advances in safe, efficient and predictable hot-water drilling, that should be adopted into future CHWD designs and operating procedures.

Here we present preferred technical options and operational scenarios necessary to successfully access deep subglacial aquatic environments cleanly using a mobile HWD system based around the existing SLE CHWD.

## 2. Clean hot-water drill

### (a) Basic design

**Figure 2.**
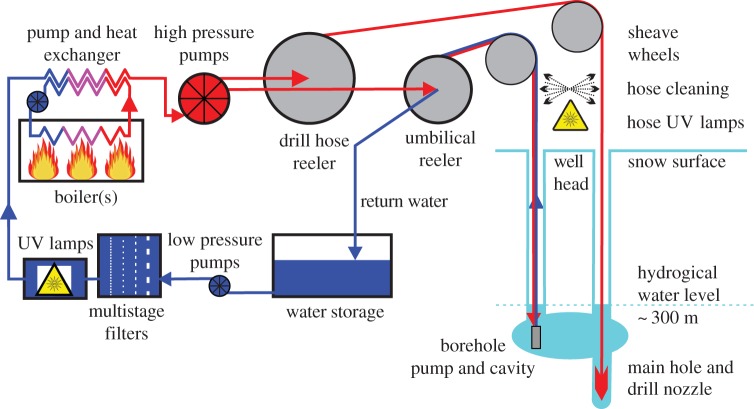
Schematic of the optimized CHWD water circulation system.

Pressurized hot water delivered down a hose and ejected at the end of a drill nozzle is a relatively simple way of delivering large amounts of energy, over long distances, to rapidly melt access holes through ice sheets. During drilling operations, the hose and drill nozzle are lowered slowly, ensuring they hang as a plumbline to form a straight vertical drill hole which the admixture of drill and melt water also uses as the return conduit. A borehole pump, installed below the hydrological level (the water level in the hole after the ice base is penetrated) in a separate but hydrologically connected hole, continually recovers water to a surface storage tank. The water is then filtered, UV-treated, heated via a heat exchanger and re-used by the HWD (figure 2).

HWD technology was developed in the 1970s and has been adopted since then by numerous groups working on various glaciers, ice streams and ice shelves in both hemispheres [13]. To date, the technique has provided a rapid and relatively cost-effective means of drilling holes in ice up to 2500 m deep and 60 cm in diameter. Drilling to access the ice sheet base beyond this depth, however, and doing so cleanly, requires the development of a new-specification CHWD.

In December 2008, funding was granted by the UK Natural Environment Research Council (NERC) for the exploration of SLE, with drill design and construction beginning late in the following year. The major CHWD technology requirements faced by the programme were to extend both the drilling capabilities to greater than 3000 m and a water recovery system to greater than 300 m, and to develop clean access drill technology and procedures. In summer 2011, the new CHWD left the UK and reached the SLE drill site in early 2012. During drilling operations in December 2012, however, a culmination of equipment failures stopped the drilling. Following this failure, the NERC commissioned a formal independent FRB to determine what had gone wrong and how it could be rectified. The FRB was supportive of the CHWD approach taken by the SLE programme [14,15], concluding that hot-water drilling is the only viable option for deep and clean access. The FRB also found that, with enhancements from other successful hot-water drilling programmes, much of the CHWD high-level requirements (table 1) and system specification (table 2) developed by the programme [16,17] remained valid for the task the system was designed to accomplish.

**Table 1.** High-level requirements for a deep-ice CHWD.

provide clean access holes, 36 cm in diameter (greater than 20 cm after 24 h), up to 3500 m deep
operate through ice with minimum temperatures of −35°C
operate at sites where gas hydrates (clathrates) may have floated to the lake surface
operate at 2000 m altitude with ambient summer temperatures of −25°C
survive winter storage temperatures of −55°C
support the clean deployment and recovery of various probes and samplers
minimize drilling time and fuel requirements
remain compatible with available logistics and limitations (IL-76, LC130 aircraft, ship cranage, sea ice operations and over snow traverse)
minimize the logistic burden

### (b) Drill specification

Building on SLE experiences [14], the SLE FRB recommendations, knowledge from other HWD projects [10,12,18], and existing SLE CHWD equipment and operating procedures [17], the basic optimized drill system characteristics are given in table 2. Critically, the drill hose size which defines the CHWD size, including the entire supporting infrastructure, and the maximum thermal power that can be delivered down the hole [13], should remain unchanged from the original SLE CHWD.

**Table 2.** Deep-drill system specification.

32 mm (1.25′′) bore drill hose, 3500 m long and self-supporting
maximum water temperature at drill hose: 90°C
maximum water pressure at pumps: 15 000 kPa
maximum flow rate to drill hose: 210 l min^−1^
thermal power to drill hose: 1.35 MW
thermal power to umbilical: 0.15 MW
maximum drill speed: 2 m min^−1^
maximum upward ream speed: 10 m min^−1^
maximum water recovery depth: 350 m
maximum boiler efficiency assumed: 85%
electrical power: greater than 150 kW
estimated fuel usage: 220 l h^−1^
minimum estimated fuel per 3500 m hole: 17 600 l

With the drill infrastructure requirements specified, system efficiency, overall fuel usage and fuel contingency remain to be defined. While minimum fuel usage estimates provide a useful indication of the fuel volumes required, actual field usage can be significantly higher, resulting from system and procedural inefficiencies or equipment failures. This can be most acute during the field commissioning phase and first use as demonstrated with IceCube's EHWD where over three times the planned fuel, totalling 102 000 l, was required to drill the first hole. In the following three field seasons, the fuel consumption reduced to approximately 28 000 l per hole. With increasing system reliability and greater experience, this figure was further reduced to 21 000 l per hole over the final three seasons, with a minimum fuel usage of 15 000 l achieved on hole 32 [12]. These figures over seven seasons clearly show how initial fuel use can be higher than anticipated, yet with growing experience and increased reliability, major gains in efficiency are achievable.

If the CHWD system specified above were to be used at sites in East Antarctica, the combination of harsher environmental conditions of higher altitude, and lower air and ice temperatures (compared with West Antarctica), would need to be accommodated in the high-level requirements. The main impact on drilling would be increased refreezing rates and, to a lesser extent, an additional power requirement in the initial melting of ice. To exemplify the issue, typically in West Antarctica, a 3500 m deep hole within ice at a temperature of −32°C, the energy loss due to refreezing would be up to 700 kW. In East Antarctica, by contrast, where minimum ice temperatures can be <−50°C, this would increase to greater than 1000 kW. As the maximum energy delivery is limited by the drill hose size, accommodating these harsher conditions using the same drill would need either slower drilling to offset the increased refreezing rate or accepting a reduced initial hole diameter with less time available for instrument deployments [19]. Using smaller diameter probes and corers could significantly offset the effects of having a smaller access hole. Based on this simple analysis, it is easy to see why drilling to subglacial lakes in West Antarctica is a far easier proposition than in East Antarctica, all other things being equal.

### (c) Clean technology requirements

The first and perhaps greatest challenge in subglacial lake access is to ensure that any life detected has originated in the system under investigation and has not merely been incorporated into samples via the access drilling and sampling procedures. Such contamination can compromise the integrity of both the retrieved sample and the subglacial environment. Advances in cleanliness protocols have been met in a number of fields including space research, medical operations, and pharmaceutical and food industries. Given these advances, and the SCAR code of conduct into subglacial research [20], it is both important and feasible that the development and use of drill and probe systems that ensure clean access and sampling are fully incorporated into subglacial access programmes.

From its inception, the SLE programme developed hardware systems and operating protocols to ensure a negligible and quantifiable level of contamination and conforming to the SCAR code of conduct. For the CHWD water cleanliness was assured by a multistage filtering down to 0.2 μm to remove particles, bacteria and some viruses, a 200 W, 254 nm, ultraviolet lamp and pasteurization to 90°C to kill any remaining microorganisms, while hose outer surfaces were cleaned with ethanol before entering the access holes [14,17]. For the SLE lake probe [21] and corer [22], cleanliness was fundamental to the initial designs and cleaning methods [21], and their deployment procedures [17], to remain compliant with the SCAR code of conduct on subglacial lake exploration.

Future re-engineering and enhancement of the SLE CHWD will utilize drill and instrumentation deployment procedures developed by the original SLE programme to meet the cleanliness standards. Testing the validity of these systems and procedures is needed, however, to optimize the procedure. For drilling, adoption of the WISSARD programme wellhead UV collar, hose washing system and medical standard compressed air for flushing pipes [10,23] can also be considered. In addition to equipment development, quantifying the drill site microbiology, both introduced and *in situ*, and its potential for introduction into the subglacial access hole is essential to maintaining cleanliness standards [24], as is testing the efficiency of the cleaning procedures, ensuring filters and decontamination procedures are fully effective.

## 3. Lessons learned and likely solutions

The SLE FRB made 45 recommendations to remedy the problems encountered during the fieldwork. A summary is given by Siegert *et al.* [14], listing the SLE failures and proposed solutions, or options to consider, in future work. A large number of the issues were minor and are relatively easily solved with existing technology as demonstrated by other successful drills [10,12,18]. These include precise reeler control, robust drill instrumentation, electrical interference suppression, use of multiple boilers and drill safety systems. Further innovative developments by other drilling groups and technology advances can also be considered within a revised fully optimized system. While it is not possible to discuss all clean drilling systems, processes and technical details, the major issues raised from the first deep drilling attempt, by the SLE programme, are given here.

### (a) Hole verticality and two-hole system

A key concern for gaining subglacial access is establishing and then maintaining a subsurface water recirculation system below the local hydrological water level, during both drilling and upward reaming operations, and after the ice base has been penetrated. On ice shelves, it is a common practice to drill two closely spaced holes (less than 1 m apart) up to around 100 m deep, interconnected by a cavity to enable drill water recirculation [18]. Using a separate hole for the umbilical and borehole pump prevents entanglement with either the drill hose or instrumentation cables. Pressure sensors located with the borehole pump also provide a critical and very clear indication of when the ice base is penetrated as the water level in the hole adjusts to the actual hydrologic level. During operations at SLE, where the hydrological depth is much deeper, this process was attempted below 300 m and with a 2 m hole separation dictated by the wellhead rail system [17]. No interconnection between the two holes was observed, and because of this drilling was halted. The SLE FRB concluded that issues in both equipment performance and the drilling procedure may have resulted in non-vertical holes, further increasing hole separation making it unlikely that an interconnecting cavity could be established. Water from the main borehole also over spilled and froze in the umbilical hole, causing the loss of the borehole pump and umbilical. This latter issue may have made it impossible to observe a hydrological connection, even if one were made. Indeed one cannot rule out the possibility that a hydrological connection was made, but not detected. Ensuring successful operation requires mitigation of these critical issues.

At IceCube, borehole position data show typical deviations from the vertical of approximately 10–20 cm, and occasionally up to 35 cm at depths of approximately 300 m when drilled under normal operating conditions and at speeds close to 2 m min^−1^. These data demonstrate that vertical holes can be drilled to over 300 m within approximately one hole radius, provided the drill system and its instrumentation are functioning as needed, although the data also show the holes to be slightly helical. To maintain the hole separation at depth, the main drill should be used to make both holes, thereby avoiding the complexities of a combined borehole pump-drill assembly [17] and the need for precision low-speed umbilical winch control, as well as the potential effects of residual curvature in the very large 11 cm diameter umbilical potentially preventing it from hanging vertically within the hole.

### (b) Cavity formation

**Figure 3.**
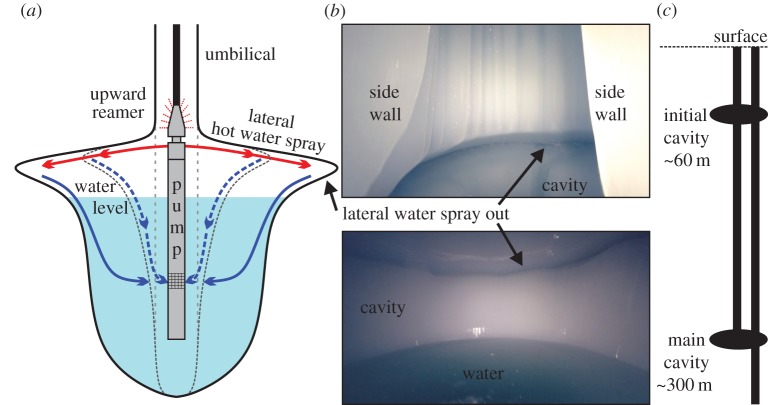
(*a*) Schematic of cavity formation, (*b*) images from inside a cavity at 90 m depth and (*c*) schematic of the proposed two-hole, two cavity system.

Provided they are drilled vertically, formation of an interconnecting cavity between two closely spaced holes (less than 0.6 m), using a lateral fan spray, is routine even in a water-filled hole [18]. However, if greater side wall penetration is required, then the lateral fan spray must be located above the borehole pump and the water level drawn down below the sprays but above the pump intake to maintain water recovery to the surface (figure 3*a*). In air, the high velocity horizontal sprays are extremely effective at penetrating deep into borehole side walls, rapidly forming a wide cavity, as confirmed by recent field tests on Ronne Ice Shelf (figure 3*b*). Using this technique with only 50% of the SLE CHWD capacity (100 l min^−1^), it is feasible to form a 4 m diameter cavity within about 20 min.

### (c) Cavity maintenance and borehole pump recovery

During subglacial lake instrument deployment (up to 24 h), freezing of the water surface at the top of the water-filled hole could easily damage instruments, and instrument cooling can lead to rapid ice accretion on contact with water. To prevent these issues from occurring, water can be recovered from the cavity, heated and returned to the cavity to maintain it around 5°C, thereby ensuring instruments are ice-free either before proceeding down to the ice base or being recovered to the surface.

Deployed at depth, into a cold water-filled environment for periods of several days, the condition of the borehole pump, its umbilical and its instrumentation must be regularly checked and maintained to prevent it becoming frozen into the borehole. Heat conduction from the hot water pumped down the relatively large 11 cm diameter umbilical cannot provide sufficient energy (300 to 600 W m^−1^) to combat hole refreezing in the water-filled hole but this problem can be minimized by drilling an oversized hole initially. As an operational necessity, the pump should occasionally oscillate between its maximum deployment depth and the water surface using the spray block to ream the hole and mitigate against the contact freezing that can occur between the umbilical and hole wall. As additional insurance against entrapment by refreezing, an upward valve activated reamer comparable with that described by Makinson & Anker [18] could be installed above the lateral spray block to allow the pump to drill its way back to the surface if necessary (figure 3*a*).

### (d) Reducing initial water loss

Seed water for the CHWD should be made by using a vehicle with a blade or scoop to lift surface snow into a melt tank. The melt water is then stored in a covered surface tank, or large insulated pillow tank to minimize surface contamination and heat loss. This reservoir acts as a buffer accommodating any temporary stoppages in the water recovery system. To minimize the significant water losses into the permeable firn during the formation of the deep (greater than 300 m) interconnected two-hole system, the technically simplest method would be to drill through the firn and establish an initial shallow interconnecting cavity and water recirculation system at about 60 m (figure 3*c*), or below the level of permeability, before proceeding with both holes to 300 m and forming the deeper interconnecting cavity. Drilling to 60 m and then to 300 m would provide a full CHWD system test before committing to deeper drilling. At each stage, the borehole pump would be deployed and the holes pumped almost dry to facilitate rapid lateral cavity formation in the air-filled hole (figure 3*a*), connecting the two holes. A combined drill and borehole pump system could be considered to make both the holes and the connecting cavity below 300 m, similar to the system used at SLE [17], and on the Askaryan Radio Array (ARA) HWD at the South Pole (T. Benson 2013, personal communication). Once the combined drill and pump system is below the firn, each hole would only have a few metres of water above the pump during the drilling, with the remainder of the hole remaining dry thus minimizing borehole refreezing. However, the added technical complexities involved make this option less desirable. During drilling down to 300 m, using either method, approximately 60 m^3^ of water could be recovered from the boreholes and stored at the surface. A substantial volume of this water (greater than 35 m^3^) is needed to replenish the approximately 10% volume loss during the main drilling phase and to maintain a constant borehole water level. In upcoming fieldwork scheduled for the 2017/18 season, the technically simpler method of having a separate drill and pump system, and forming an initial cavity at 60 m before creating a deeper cavity, will be implemented at multiple sites when drilling greater than 2000 m on Rutford Ice Stream for subglacial sediment access. Here two borehole pumps will be installed, each in their own hole, in a deep cavity up to 280 below the surface.

### (e) Reeler and drill tower

Precise reeler control and accurate level wind on and off the reels are essential for efficient drilling. Reelers can be powered either hydraulically or electrically and although hydraulic systems can be used to prevent problems with electrical noise, some implementations have suffered from problems with large temperature fluctuations adversely affecting fluid viscosity, low torque at slow speeds and poor positional accuracy, though these features can be overcome with appropriate design. Currently, most HWDs use electrical variable frequency drives (VFDs) [12,18,25] as standard. The SLE reelers also took the full tension of the hose or umbilical, resulting in diameter changes that were problematic for the level wind mechanism. To alleviate this problem, a capstan [18] or linear traction drive [10] could be installed together with an accurate level wind mechanism [26] to aid the precision of the winching process.

Essential to the rapid interchange of the drill and borehole pump between holes, and rapid deployment of probes and corers, is a new multipurpose tower. Increased headroom between the sheave wheels and the snow surface would greatly improve equipment deployment and recovery times and reduce the reliance on a hydraulic crane. Similarly, probe and corer deployment times could be also be reduced significantly if they were fully prepared for immediate deployment on the tower as the hole became available.

## 4. Drill instrumentation and monitoring

Reliable drill instrumentation, communications and monitoring systems are essential for safe and successful hot-water drilling. Key parameters critical to the drilling process such as water temperature, flow rate, drill speed, depth and load, and hole water level must be reliable and have built-in redundancy, including simple mechanical gauges as well as digital sensors with local readouts and adequate spares. Large HWDs such as those used for WISSARD and IceCube have operated successfully in Antarctica with varying levels of monitoring, centralized control and manual control. These complex drills can have many tens of VFD motors and hundreds of sensors or actuators, although a drawback of VFDs is their output of electrical noise. However, appropriately sized line-reactors for each motor will minimize noise and for signal cables, shielding and filters will block the noise effectively [12,25].

Down-hole instrumentation just behind the drill nozzle is rarely used on HWDs. Occasionally, standalone instruments are used to measure hole water temperature and pressure above the nozzle, and drill water temperature arriving at the nozzle. Once drilling is complete, the data can be used to confirm drill hose thermal conductivity and stretch, and to calculate hole evolution above the nozzle. The EHWD used a fully instrumented drill nozzle that measured these parameters, including hole inclination and diameter, with live data streamed to the surface via a dedicated communications cable taped to the drill hose. These data confirmed drill performance, verticality and hole diameter that was particularly important for adjusting upward ream speeds to attain the required final hole diameter [12]. However, the EHWD method of data transmission does not lend itself easily to clean drilling operations, whereas wires embedded in the hose or a system based on the wireless subglacial sensor system (WiSe) [27], or acoustic data transmission, may offer viable communications alternatives but have yet to be assessed for clean deep drilling operations.

New CHWD instrumentation, communications and monitoring systems (both surface and down hole) are currently being developed by BAS. During forthcoming polar drilling campaigns testing and development of reliable instrumentation and communications systems will continue.

## 5. Integrated drill prediction model

**Figure 4.**
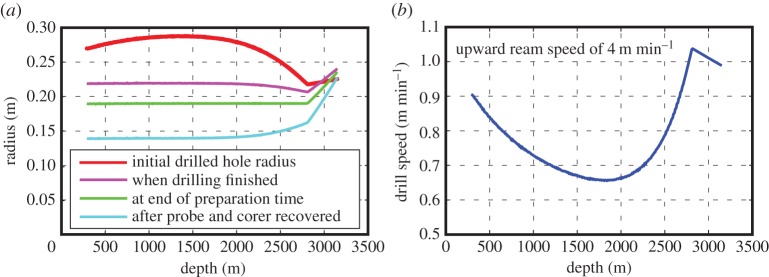
Example plots of (*a*) borehole radius a various times during and after drilling, and (*b*) the corresponding drilling speed required to create an access hole into SLE through minimum ice temperatures of −32°C. The water level is at 290 m in the hole and the drill receives 200 l min^−1^ of water at 90°C at the surface.

Having a good understanding of how a borehole diameter evolves during drilling and reaming, and the rate at which it refreezes, is essential if the hole is to remain large enough for the safe deployment and recovery of subglacial probes and samplers. To this end, a model of the drilling procedure has been used to determine the rate of drilling and hole evolution and is based on field data [28] and theory of heat and mass exchange [29]. The ultimate aim is to drill a hole that is vertical and has a specified uniform diameter along its length at a specified time in the future (i.e. during instrument deployments). Using input parameters such as drill water temperature and flow rate, ice temperature profile, time-dependent freezing rate, drill hose thermal conductivity and maximum permissible drilling rates, the model provides a drill speed depth profile assuming a fixed upward ream speed. The total time taken for drilling and reaming enables the idealized fuel usage to be calculated, excluding contingencies. Figure 4 shows the empirical model predictions applicable to an SLE-type borehole at various times during and after the drilling and reaming. A detailed description of a physics-based model focusing on borehole size, lifetime and fuel consumption is given by Greenler *et al.* [19]. With the borehole size prescribed, the drill speed depth profile from the model is defined by the operational parameters, primarily water temperature and flow rate. Deviations from this drilling profile, because of breakdowns, for example, can be corrected by modifying the drilling or reaming speeds to maintain the future target diameter. By integrating the real-time drilling data into the model, adjustment of the upward ream speed profile would be automatic and changes in drill system performance could also be accounted for. Furthermore, if real-time down-hole diameter measurements acquired during the upward ream are available, then a precise rather than a predicted ream speed could be used to better guarantee the required final hole diameter [12]. Borehole diameter measurement devices, based on mechanical, optical or acoustic systems, built into probes and sediment corers, could monitor the narrowing of the hole as it refreezes, minimizing the chances of instruments becoming trapped, while enabling maximum use of the hole.

## 6. Drill performance

The maximum thermal power that can be delivered to the drill nozzle is dependent on the hose bore and length. In overcoming the frictional losses in the hose that increase with flow velocity squared [18], the practical pressure limit of 15 000 kPa for standard commercial equipment is soon reached. For example, for the same thermal power system, increasing the hose bore from 32 mm to 38 mm would decrease the operating pressure, pumping power, and electrical power generation requirements by approximately 60%. However, the larger size and increased weight of a continuous drill hose, reeler and supporting infrastructure would be increased by at least 50%, making it incompatible with available logistics. Thus, an optimal balance must be determined.

With the maximum HWD power defined, significant operating efficiencies in terms of minimizing drilling time and fuel usage, while maximizing hole availability, are essential for any deep drilling programme and can be gained from system reliability, predictability and accumulated experience [12]. Rigorous testing of any new drill system is also vital for reducing risk. Significant efficiencies can be gained from reductions in hole diameter and by using smaller instruments. As fuel consumption and drilling time are approximately proportional to the square of the hole diameter [19], reducing the 36 cm diameter by just 10% reduces the predicted minimum fuel consumption by about 20%, or at least 3500 l. Similar efficiencies can be gained by reducing procedural times during the science phase, such as minimizing handling, cleaning, and connecting times. In addition, by using smaller diameter instruments, hole usage and the subglacial science activities are significantly increased. Furthermore, reaming of the hole would extend the access time, assuming fuel availability and a viable drill water recirculation system. However, for any access hole and its utilization there is always a balance between fuel conservation and the risk of an inadequately sized hole [19], hence safe working clearances must be predefined based on predicted refreezing rates. If, however, each probe and corer has hole diameter measuring capabilities, tolerances could be reduced, enhancing efficiencies further.

## 7. Logistics and mobility

Compatibility of the CHWD with a range of available logistics and operational limitations (table 1) are primary design parameters. The SLE CHWD, probes, winches and entire camp infrastructure, weighing around 130 T, with fuel (51 250 l) and sleds constituting a further 50 T, were deployed entirely by aircraft and over snow traverse. Future drill modifications and enhancements will almost certainly increase overall weight of the drill system, increasing the logistic burden. Investing in new low friction sled technologies could accommodate this probable weight increase without increasing the logistics fuel requirement. For example, the greater use of plastic sleds, fuel bladders and, for ISO containers, the use of either thick foam interfaces on plastic sleds or Air Ride Cargo Sleds (ARCS) [30], could reduce towing loads significantly. With PistenBully® vehicles generally able to tow around 50 T but probably not more than 60 T, it should be possible to limit the total number of drill deployment vehicle journeys to three or four when including the overhead of the traverse fuel and accommodation caboose. Having dedicated on-site vehicles also allows easy movement of the drill if initial problems are encountered with the access holes or if more than one hole per season is attempted. With a reliable CHWD and optimized drilling strategy attaining minimum drill fuel usage, it would be possible to drill a 36 cm hole, 3500 m deep, using less than 20 000 l. For the field commissioning and initial use, however, more fuel is likely to be needed [12], and a significant contingency is required at the first drill site.

## 8. Outstanding issues

Unlike the deep hot-water drilling at IceCube, which became routine, future clean access drilling is likely to take place at widely differing locations, presenting additional technical complexities and uncertainties. For highly efficient drilling and accurate prediction of borehole integrity, the ice temperature profile is needed to determine both the energy required to melt the ice and, more importantly, the rate of hole closure from refreezing. There can be significant differences in borehole freeze rates and other factors between sites. For example, in localized high melt rate areas the increased vertical advection, which can be seen by the dipping layers in radargrams, draws down colder ice, reducing the mean ice column temperature. As other processes can have similar effects, ice sheet models or recent seismic methods [31] are needed at each new site to minimize uncertainties in the ice temperature profile. Similarly, the ice age-depth profile can be used to determine the proportion of ice from last glacial period: this contains relatively high levels of 1–3 μm sized dust, which will eventually reach the surface filters during the drilling process and affect the predicted filter lifetimes.

Further uncertainties are present on reaching the ice base. Potentially, high concentrations of sediment in basal ice can slow the drilling process but the use of a high velocity drill water jet, fluidising the sediments, can overcome significant volumes of sediment until, in extreme cases, a collection device attached to the drill nozzle is required to remove sediment from the hole [18]. Also, if large volumes of clathrate have floated to the lake surface, heat from the drilling process can be destabilizing, causing rapid degassing and a potentially dangerous blowout of water and gas from the borehole [32]. At each site, an assessment of potential clathrate build up will be needed, but if uncertainly remains then cold drilling (less than 5°C) at the ice base the hole will mitigate the potential dangers of blowout.

On reaching the ice base, the water in the borehole will adjust to a level that reflects the subglacial water pressure. Accurately predicting this level is critical for determining the cavity depth and maintaining the drill water recirculation system after accessing the bed. The loss of water from the cavity would seriously compromise later science activities by preventing the upward reaming of the hole, limiting its size and lifetime. Away from the margins of subglacial lakes, hydrostatic equilibrium can be assumed and the hydrological level is relatively easy to determine, provided no significant vertical scale topography is present and no ice bridging occurs. In regions where ice is grounded and the basal hydrology forms part of an extended drainage network, the local hydrological level is likely to be uncertain by tens of metres [33]. Modelling of the subglacial hydrology will be needed to reduce uncertainties and ensure the cavity and borehole pump are placed below the hydrological level.

For any deep-ice drilling campaign, having sufficient flexibility within the logistics, the drilling equipment and the operational procedures is often the key to its success. A further key aspect is having adequate numbers of experienced field personnel familiar with all aspects of field operations and the drilling system, a point highlighted by the SLE FRB [14]. Clearly, any future deep drilling projects must provide sufficient training and staffing to support the drilling operations.

## 9. Conclusion

Clean hot-water drilling remains the most viable technique for directly accessing the vast subglacial environment beneath the East and West Antarctic Ice Sheets, especially over regions where water is present at the bed. Hot-water drilling has a proven track record to depths of 2500 m and cleanly to 800 m, but the first attempt to extend clean drilling to greater than 3000 m at SLE was unsuccessful. Despite this setback, the lessons learned are now well understood. Building on these experiences and those of other ongoing drilling programmes, together with external review advice, alternative and emerging drilling equipment, tools and techniques are being investigated, developed and tested in preparation for the re-engineering and enhancement of the revised SLE CHWD. Comprehensive reviews of the system design and procedures, combined with detailed testing and evaluation, will eliminate most of the technical issues experienced at SLE. If the ultimate goal of drilling deep and clean subglacial access holes in a safe, efficient and predictable way is to be achieved routinely, then a thorough design optimization review is required, including bench testing and field trials.

## References

[1] Wright A, Siegert M 2012 A fourth inventory of Antarctic subglacial lakes. Antarct. Sci. 24, 659–664. (10.1017/S095410201200048X)

[2] Pattyn F 2010 Antarctic subglacial conditions inferred from a hybrid ice sheet/ice stream model. Earth Planet. Sci. Lett. 295, 451–461. (10.1016/j.epsl.2010.04.025)

[3] Van Liefferinge B, Pattyn F 2013 Using ice-flow models to evaluate potential sites of million year-old ice in Antarctica. Clim. Past 9, 2335–2345. (10.5194/cp-9-2335-2013)

[4] Ashmore DW, Bingham RG 2014 Antarctic subglacial hydrology: current knowledge and future challenges. Antarct. Sci. 26, 758–773. (10.1017/s0954102014000546)

[5] Livingstone SJ, Clark CD, Woodward J, Kingslake J 2013 Potential subglacial lake locations and meltwater drainage pathways beneath the Antarctic and Greenland ice sheets. Cryosphere 7, 1721–1740. (10.5194/tc-7-1721-2013)

[6] Wright AP *et al.* 2012 Evidence of a hydrological connection between the ice divide and ice sheet margin in the Aurora Subglacial Basin, East Antarctica. J. Geophys. Res. 117, F01033 (10.1029/2011jf002066)

[7] Bentley CR, Koci BR 2007 Drilling to the beds of the Greenland and Antarctic ice sheets: a review. Ann. Glaciol. 47, 1–9. (10.3189/172756407786857695)

[8] Lukin VV, Vasiliev NI 2014 Technological aspects of the final phase of drilling borehole 5G and unsealing Vostok Subglacial Lake, East Antarctica. Ann. Glaciol. 55, 83–89. (10.3189/2014AoG65A002)

[9] Lipenkov VY, Ekaykin AA, Polyakova EV, Raynaud D 2016 Characterization of subglacial Lake Vostok as seen from physical and isotope properties of accreted ice. Phil. Trans. R. Soc. A 374, 20140303 (10.1098/rsta.2014.0303) 26667912

[10] Rack FR, Duling D, Blythe D, Burnett J, Gibson D, Roberts G, Carpenter C, Lemery J, Fischbein S 2014 Developing a hot-water drill system for the WISSARD project: 1. Basic drill system components and design. Ann. Glaciol. 55, 287–297. (10.3189/2014AoG68A031)

[11] Rack FR 2016 Enabling clean access into Subglacial Lake Whillans: development and use of the WISSARD hot water drill system. Phil. Trans. R. Soc. A 374, 20140305 (10.1098/rsta.2014.0305) 26667915

[12] Benson T *et al.* 2014 Icecube enhanced hot water drill functional description. Ann. Glaciol. 55, 105–114. (10.3189/2014AoG68A032)

[13] Bentley CR *et al.* 2009 Ice drilling and coring. In Drilling in extreme environments, pp. 221–308. Weinheim, Germany: Wiley-VCH, Weinheim.

[14] Siegert MJ, Makinson K, Blake D, Mowlem M, Ross N 2014 An assessment of deep hot-water drilling as a means to undertake direct measurement and sampling of Antarctic subglacial lakes: experience and lessons learned from the Lake Ellsworth field season 2012/13. Ann. Glaciol. 55, 59–73. (10.3189/2014AoG65A008)

[15] Schiermeier Q 2014 Polar drilling problems revealed. Nature 505, 463 (10.1038/505463a) 24451520

[16] Siegert MJ *et al.* 2007 Exploration of Ellsworth Subglacial Lake: a concept paper on the development, organisation and execution of an experiment to explore, measure and sample the environment of a West Antarctic subglacial lake. Rev. Environ. Sci. Biotechnol. 6, 161–179. (10.1007/s11157-006-9109-9)

[17] Siegert MJ *et al.* 2012 Clean access, measurement, and sampling of Ellsworth Subglacial Lake: a method for exploring deep Antarctic subglacial lake environments. Rev. Geophys. 50, RG1003 (10.1029/2011rg000361)

[18] Makinson K, Anker PGD 2014 The BAS ice-shelf hot-water drill: design, methods and tools. Ann. Glaciol. 55, 44–52. (10.3189/2014AoG68A030)

[19] Greenler L, Benson T, Cherwinka J, Elcheikh A, Feyzi F, Karle A, Paulos R 2014 Modeling hole size, lifetime and fuel consumption in hot-water ice drilling. Ann. Glaciol. 55, 115–123. (10.3189/2014AoG68A033)

[20] ATCM. 2011 Final Report of the Thirty-fourth Antarctic Treaty Consultative Meeting, p. 348. Buenos Aires, Argentina, 20 June–1 July 2011. Buenos Aires, Argentina: Secretariat of the Antarctic Treaty.

[21] Mowlem MC *et al.* 2011 Probe technology for the direct measurement and sampling of Ellsworth Subglacial Lake. In Antartic subglacial aquatic environments (eds MJ Siegert, MC Kennicutt II, R Bindschadler), pp. 159–186. Washington, DC: AGU.

[22] Hodgson DA *et al.* 2016 Technologies for retrieving sediment cores in Antarctic subglacial settings. Phil. Trans. R. Soc. A 374, 20150056 (10.1098/rsta.2015.0056) 26667918

[23] Priscu JC *et al.* 2013 A microbiologically clean strategy for access to the Whillans Ice Stream subglacial environment. Ant. Sci. 25, 637–647. (10.1017/s0954102013000035)

[24] Pearce DA, Magiopoulos I, Mowlem M, Tranter M, Holt G, Woodward J, Siegert MJ 2016 Microbiology: lessons from a first attempt at Lake Ellsworth. Phil. Trans. R. Soc. A 374, 20140291 (10.1098/rsta.2014.0291) 26667906

[25] Burnett J *et al.* 2014 Developing a hot-water drill system for the WISSARD project: 3. Instrumentation and control systems. Ann. Glaciol. 55, 303–310. (10.3189/2014AoG68A039)

[26] Mortensen NB, Johnson JA, Shturmakov AJ 2014 Precision cable winch level wind for deep ice-coring systems. Ann. Glaciol. 55, 99–104. (10.3189/2014AoG68A013)

[27] Smeets CJPP, Boot W, Hubbard A, Pettersson R, Wilhelms F, van den Broeke MR, van de Wal RSW 2012 A wireless subglacial probe for deep ice applications. J. Glaciol. 58, 841–848. (10.3189/2012JoG11J130)

[28] Makinson K 1993 The Bas hot-water drill—development and current design. Cold Reg. Sci. Tech. 22, 121–132. (10.1016/0165-232X(93)90051-9)

[29] Humphrey N, Echelmeyer K 1990 Hot-water drilling and bore-hole closure in cold ice. J. Glaciol. 36, 287–298. (10.3189/002214390793701354)

[30] Lever JH, Weale JC 2012 High efficiency fuel sleds for polar traverses. J. Terramech. 49, 207–213. (10.1016/j.jterra.2012.05.001)

[31] Peters LE, Anandakrishnan S, Alley RB, Voigt DE 2012 Seismic attenuation in glacial ice: a proxy for englacial temperature. J. Geophys. Res. 117, F02008 (10.1029/2011jf002201)

[32] Brito MP, Griffiths G, Mowlem M, Makinson K 2013 Estimating and managing blowout risk during access to subglacial Antarctic lakes. Antarct. Sci. 25, 107–118. (10.1017/s0954102012000442)

[33] Engelhardt H, Kamb B 1997 Basal hydraulic system of a West Antarctic ice stream: constraints from borehole observations. J. Glaciol. 43, 207–230.

